# Anxiety disorders: Psychiatric comorbidities and psychosocial stressors among adult outpatients

**DOI:** 10.4102/sajpsychiatry.v24i0.1138

**Published:** 2018-05-24

**Authors:** Carla Nel, Linda Augustyn, Nandie Bartman, Marizél Koen, Maggy Liebenberg, Jurgens Naudé, Gina Joubert

**Affiliations:** 1 Department of Psychiatry, University of the Free State, South Africa; 2 School of Medicine, University of the Free State, South Africa; 3 Department of Biostatistics, University of the Free State, South Africa

## Abstract

**Background:**

Anxiety disorders are the most prevalent class of lifetime mental disorders according to South African research. However, little is known about the prevalence of factors that might complicate treatment among adults in a psychiatric outpatient setting.

**Aim:**

To explore the psychiatric comorbidities and psychosocial stressors among a population of adults treated for anxiety disorders at the outpatient unit of a tertiary psychiatric facility in Bloemfontein.

**Methods:**

In this retrospective cross-sectional study, clinical files of all mental healthcare users receiving treatment were reviewed to identify those with a current or previous diagnosis of one or more of the following anxiety disorders: generalised anxiety disorder (GAD), panic disorder, social anxiety disorder (SAD) and agoraphobia.

**Results:**

Of the 650 available records, 103 (15.8%) included at least one anxiety disorder. Of those, 65.1% had GAD, 34.0% had panic disorder and 29.1% had SAD. Agoraphobia was diagnosed in 14.6% of patients almost exclusively as comorbid with panic disorder. Additional psychiatric disorders were present for 98.1% of patients and 36.9% had multiple anxiety disorders. The patients had a history of relational problems (64.1%), educational and occupational stressors (55.3%), abuse and neglect (28.2%), other problems related to the social environment (24.3%) and self-harm (23.3%).

**Conclusion:**

Clinical practice should take the high rates of comorbidity into account and the importance of integrated substance-related interventions in mental healthcare settings is clear. Diagnostic practices regarding agoraphobia without panic, and the comorbidity of anxiety and personality disorders should receive further attention. Clinicians should be aware of the potential impact of the frequently reported psychosocial stressors.

## Introduction

As part of the South African Stress and Health (SASH) study, which investigated the lifetime prevalence of common mental disorders, anxiety disorders were found to be the most prevalent class of lifetime mental disorders (15.8%), followed by substance use disorders (13.3%) and mood disorders (9.8%).^1^ The SASH study revealed that agoraphobia without panic was one of the three most prevalent individual lifetime mental disorders at 9.8%, along with alcohol abuse (11.4%) and major depressive disorder (9.8%). Anxiety disorders had the highest 12-month prevalence (8.1%) of all psychiatric disorders, and the Free State’s provincial lifetime prevalence of anxiety disorders was significantly higher than countrywide rates.

The presence of severe depression or anxiety disorders has been associated with significantly reduced earnings among employed and unemployed South African adults.^2^ Data from the SASH study on the association of mental and physical disorders with days out-of-role (i.e. unable to work or carry out day-to-day activities) indicated that the highest reported days out-of-role were associated with mental disorders (28.2 for anxiety and 27.2 for depression), followed by physical disorders (24.7 for arthritis and 21.7 for pain). The presence of any anxiety disorder was estimated to have the highest individual-level effect, with 12.7 days more per year of being out-of-role compared to those estimated for the average individual without the disorder.^1,3^ These associations were drawn from a cross-sectional study and, therefore, do not imply causality.

The treatment of anxiety disorders may be complicated by the potential presence of comorbid psychiatric disorders, including other anxiety disorders, mood disorders, substance use disorders and personality disorders.^4,5,6,7^ In addition to comorbidity, psychosocial stressors and behaviour such as self-harm^7^ may require clinical attention as they have the potential to affect course, prognosis or treatment of the disorder.^8^ Because much of what is known about the patterns of comorbidities of anxiety disorders is based on either international studies or information gathered through research conducted on the South African general population, little is known about the potentially complicating comorbidities of anxiety disorders as they present locally in patients in a tertiary psychiatric setting, or about the psychosocial challenges these patients face.

### Aim

The primary objective of this study was to explore the psychiatric comorbidities present among adults at a tertiary psychiatric facility diagnosed with one or more anxiety disorders. The secondary objective of the study was to explore the psychosocial problems reported by these mental healthcare patients.

## Research methods and design

### Study design

This was a retrospective cross-sectional study.

### Setting

The study was conducted at the adult outpatient unit at the Free State Psychiatric Complex (FSPC) in Bloemfontein, a specialist psychiatric facility in an urban setting where tertiary-level care is provided.

### Study population and sampling strategy

Routine diagnostic procedure at the FSPC outpatient unit entails that all preliminary *Diagnostic and Statistical Manual of Mental Disorders*, fifth edition (DSM-5)^8^ diagnoses made after the psychiatric intake are subject to review during multidisciplinary team rounds, overseen by a consultant psychiatrist and clinical psychologists. Clinical files of all 650 adult mental healthcare patients, who had active files during the study period from August to October 2015, at the outpatient unit were checked to identify patients with a current or previous diagnosis of one or more of the following anxiety disorders: generalised anxiety disorder (GAD), panic disorder, social anxiety disorder (SAD) and agoraphobia. Files were regarded as active if the patient had attended at least one outpatient consultation during the 6 months prior to the commencement of the study. All clinical files in which one or more anxiety disorder diagnoses were noted were selected for further data collection.

The criterion for inclusion was a recorded anxiety disorder diagnosis of GAD, panic disorder, SAD or agoraphobia. Files in which this diagnosis was last documented prior to 01 January 2006 were excluded. This was done to ensure that the documentation of the anxiety disorder diagnosis, psychiatric comorbidities and psychosocial stressors would be accessible in the clinical files, as information older than 10 years is often archived.

### Data collection

Clinical data in the patient files from all previous psychiatric contacts relevant to the study objectives were recorded on data forms. This included demographic information, diagnoses of anxiety disorders and other psychiatric disorders diagnosed concurrently with an anxiety disorder, to ensure that only diagnostic comorbidities were reflected and not all the differential diagnoses considered over the course of treatment. For clinical files in which an anxiety disorder was last recorded prior to 2013, DSM-IV^9^ diagnoses were identified and captured by the researchers according to the corresponding DSM-5^8^ classification. Psychosocial stressors reported during any of the previous psychiatric consultations during which an anxiety disorder diagnosis was made were captured by the researchers. Included in our data form were the main categories of psychosocial stressors and all other conditions that could be a focus of clinical attention study as classified in the DSM-5,^8^ including circumstances of personal history such as self-harming behaviour.

Anxiety disorders could not be classified as either the principal or the comorbid diagnosis, as this is not routinely or consistently documented in the clinical files at the FSPC outpatient unit. Patients with complex or treatment-resistant disorders are often referred with multiple psychiatric diagnoses. As noted by Norton and Chase,^10^ the classification of a disorder being principal or comorbid may be problematic, even under ideal clinical circumstances.

### Pilot study

A pilot study was conducted to test the usability of the data form, which consisted of 25 patient files selected through systematic random sampling of the 103 files that met the inclusion and exclusion criteria. Data from the pilot study were included in the main study.

### Data analysis

Data were analysed by the Department of Biostatistics, Faculty of Health Sciences, University of the Free State (UFS), using SAS Version 9.2 (SAS Institute, Cary, NC, USA). Results are summarised by frequencies and percentages (categorical variables) and medians (numerical variables because of skew distributions). The three main single diagnoses were compared statistically regarding demographic characteristics, comorbid psychiatric disorders and psychosocial stressors using Fisher’s exact tests (categorical variables) and Kruskall-Wallis tests (numerical variables). Patients with single anxiety diagnosis were similarly compared to patients with multiple anxiety diagnoses. A *p*-value of 0.05 or less was considered statistically significant.

### Ethical consideration

The study was approved by the Ethics Committee of the Faculty of Health Sciences, University of the Free State (STUD Nr 23/2015) and the Free State Department of Health. Permission to access patient files was obtained from the Ethical and Research Committee of the Free State Psychiatric Complex. Patient files were not removed from the site.

## Results

Of the 650 files of active patients at the FSPC outpatient unit, 103 (15.8%) had at least one current or past anxiety disorder on record. Of these patients, 73.8% (*n* = 76) were females and 83.5% (*n* = 86) were under the age of 60 years. The majority of patients (85.4%) resided in the Mangaung Metropolitan district, which includes Bloemfontein and the surrounding areas. Most of the patients (59.2%) had completed at least a secondary-level education and two-thirds were reported being unemployed (Table 1).

**TABLE 1:** Demographic information of the study sample (*n* = 103).

Variables	*n*	%
**Age range (years)**		
Young adulthood (18–39)	40	38.8
Middle adulthood (40–59)	46	44.7
Late adulthood (60 +)	17	16.5
**Gender**		
Male	27	26.2
Female	76	73.8
**Marital status**		
Single	40	38.8
Married	36	35.0
Widowed/separated/divorced	27	26.2
**Level of education**		
None	3	2.9
Primary	5	4.9
Some secondary schooling	34	33.0
Completed secondary	38	36.9
Tertiary	23	22.3
**Employment status**		
Working: formal employment	23	22.3
Working: informal sector	11	10.7
Non-working: unemployed	34	33.0
Non-working: student	7	6.8
Non-working: grant or pension	28	27.2

### Diagnostic morbidity and comorbidities

As shown in Table 2, the anxiety disorder diagnosed with the highest frequency was GAD (65.1%), followed by panic disorder (34.0%) and SAD (29.1%). Agoraphobia was diagnosed in 14.6% of the study sample. A large proportion of patients diagnosed with an anxiety disorder (36.9%) presented with multiple anxiety disorders, the most common combinations being GAD with SAD (11.7%) and GAD with panic disorder (9.7%). Generalised anxiety disorder was the most common anxiety disorder diagnosed as single disorder (37.9% of the study population) and occurred more frequently as a single anxiety disorder diagnosis. Panic disorder, SAD and agoraphobia were all more frequently diagnosed concurrently with other anxiety disorders than as single anxiety disorders. From the 15 patients diagnosed with agoraphobia, only one was diagnosed with agoraphobia as a single anxiety diagnosis.

**TABLE 2:** Diagnostic morbidity and psychiatric comorbidity of anxiety disorders.

Diagnoses	*n*	%
**Anxiety disorder diagnosis**		
Generalised anxiety disorder	67	65.1
• As a single anxiety disorder	39	58.2
• Concurrent with other anxiety disorders	28	41.8
Panic disorder	35	34.0
• As a single anxiety disorder	14	40.0
• Concurrent with other anxiety disorders	21	60.0
Social anxiety disorder	30	29.1
• As a single anxiety disorder	11	37.7
• Concurrent with other anxiety disorders	19	63.3
Agoraphobia	15	14.6
• As a single anxiety disorder	1	6.7
• Concurrent with other anxiety disorders	14	93.3
**Comorbid diagnosis**		
None	2	1.9
Depressive disorders	79	76.7
Substance-related and addictive disorders	37	35.9
Bipolar and related disorders	23	22.3
Trauma- and stressor-related disorders	20	19.4
Schizophrenia spectrum and other psychotic disorders	6	5.8
Somatic symptom and related disorders	6	5.8
Other anxiety disorders (e.g. specific phobia)	5	4.9
Sleep-wake disorders	4	3.9
Neurodevelopmental disorders	3	2.9
Obsessive-compulsive and related disorders	3	2.9
Personality disorders	1	1.0

Almost all of the patients (98.1%) also had another psychiatric diagnosis concurrent with the anxiety disorder on record. Depressive disorder was the most frequent diagnosed comorbidity (76.7%), followed by substance-related and addictive disorders (35.9%), bipolar and related disorders (22.3%) and trauma- and stressor-related disorders (19.4%) (Table 2). No comorbidity with any of the following diagnostic categories was found: dissociative disorders, feeding and eating disorders, elimination disorders, sexual dysfunctions, gender dysphoria, disruptive, impulse-control and conduct disorders, neurocognitive disorders and paraphilic disorders.

The psychiatric comorbidities of patients diagnosed with a single anxiety disorder and those who suffered from multiple anxiety disorders are presented in Table 3. Two demographic variables – gender and age – are also presented for these two groups. The three single anxiety disorder groups differed significantly regarding gender distribution (*p* = 0.03); there were significantly more women in the GAD and panic disorder groups compared to the SAD group. The three groups also differed significantly regarding age (*p* < 0.01), with SAD patients having the lowest median age (32 years) and the panic disorder patients with the highest (56 years). As far as comorbid psychiatric disorders are concerned, significant differences were observed regarding bipolar and related disorders (*p* = 0.05) and other anxiety disorders (*p* = 0.05). Bipolar and related disorders occurred in more than a quarter of patients with GAD and patients with SAD, but in none of the patients with panic disorder. Other anxiety disorders occurred in 21.4% of patients with panic disorder, but in only 2.6% of patients with GAD and none of the patients with SAD.

**TABLE 3:** Age, gender and diagnostic comorbidities of patients with single anxiety diagnosis or multiple anxiety diagnoses.

Variables	Generalised anxiety disorder (*n* = 39)	Panic disorder (*n* = 14)	Social anxiety disorder (*n* = 11)	*p*	Total single anxiety disorders (*n* = 65)†	Total multiple anxiety disorders (*n* = 38)	*p*
	*n* (%)	*n* (%)	*n* (%)		*n* (%)	*n* (%)	
**Demographic information**							
Median age (years)	47	56	32	< 0.01*	47	45	0.35
Male	6 (15.4)	3 (21.4)	6 (54.5)	0.03*	15 (23.1)	12 (31.6)	0.34
Female	33 (84.6)	11 (78.6)	5 (45.5)		50 (76.9)	26 (68.4)	
**Comorbid psychiatric disorders**							
No diagnosis	0 (0.0)	0 (0.0)	0 (0.0)	-	0 (0.0)	2 (5.3)	0.13
Depressive	34 (87.2)	9 (64.3)	8 (72.7)	0.15	52 (80.0)	27 (71.1)	0.30
Bipolar and related	11 (28.2)	0 (0.0)	3 (27.3)	0.05*	14 (21.5)	9 (23.7)	0.80
Substance-related and addictive	9 (23.1)	5 (35.7)	4 (36.4)	0.53	18 (27.7)	19 (50.0)	0.02*
Trauma- and stressor-related	8 (20.5)	4 (28.6)	0 (0.0)	0.15	12 (18.5)	8 (21.1)	0.75
Other anxiety (e.g. specific phobia)	1 (2.6)	3 (21.4)	0 (0.0)	0.05*	4 (6.2)	1 (2.6)	0.65
Schizophrenia spectrum and other psychotic	1 (2.6)	1 (7.1)	2 (18.2)	0.10	4 (6.2)	2 (5.3)	1.00
Neurodevelopmental	1 (2.6)	2 (14.3)	0 (0.0)	0.19	3 (4.6)	0 (0.0)	0.29
Somatic symptom and related	1 (2.6)	2 (14.3)	0 (0.0)	0.19	3 (4.6)	3 (7.9)	0.67
Obsessive-compulsive and related	1 (2.6)	0 (0.0)	0 (0.0)	1.00	1 (1.5)	2 (5.3)	0.55
Sleep-wake	0 (0.0)	0 (0.0)	0 (0.0)	0.39	1 (1.5)	3 (7.9)	0.14
Personality	0 (0.0)	0 (0.0)	1 (9.1)	0.17	1 (1.5)	0 (0.0)	1.00

† , One case of agoraphobia included.

* Statistically significant difference.

As can be seen from the results, no anxiety disorder occurred in isolation, as the two patients who did not have other psychiatric comorbidities on record had both been diagnosed with multiple anxiety disorders. The most frequently occurring psychiatric comorbidity, namely, depressive disorders, showed similar rates of occurrence in both the single and multiple anxiety disorder groups. Patients with multiple anxiety disorders had significantly greater rates of comorbid substance-related and addictive disorders compared to patients with a single anxiety disorder diagnosis (*p* = 0.02). No other significant differences were found between single and multiple anxiety diagnoses patients. Disorders, which occurred with comparable frequency between these two groups, were bipolar and related disorders (21.5% in the single anxiety disorder group compared to 23.7% in the multiple anxiety disorder group) and trauma- and stressor-related disorders (18.5% and 21.1% for the respective groups).

### Psychosocial stressors

Table 4 summarises the types of psychosocial stressors as indicated by the presence of conditions and problems that constituted a focus of clinical attention or were deemed to have an effect on the diagnosis, course, prognosis or treatment of the diagnosed anxiety disorder. As the DSM-5^8^ was used as a framework for the classification of these stressors and problems, behaviour such as self-harm was also included.

**TABLE 4:** Psychosocial stressors for patients with a single anxiety diagnosis or multiple anxiety diagnoses.

Psychosocial stressors	Generalised anxiety disorder (*n* = 39)	Panic disorder (*n* = 14)	Social anxiety disorder (*n* = 11)	*p*	Total single anxiety disorders (*n* = 65)	Total multiple anxiety disorders (*n* = 38)	*p*
	*n* (%)	*n* (%)	*n* (%)		*n* (%)	*n* (%)	
Relational problems	28 (71.8)	8 (57.1)	6 (54.5)	0.43	43 (66.2)	23 (60.5)	0.57
Educational and occupational problems	22 (56.4)	6 (42.9)	8 (72.7)	0.38	37 (56.9)	20 (52.6)	0.67
Abuse and neglect	10 (25.6)	4 (28.6)	3 (27.3)	1.00	17 (26.2)	12 (31.6)	0.55
Other problems related to the social environment	9 (23.1)	0 (0.0)	5 (45.5)	0.02*	14 (21.5)	11 (28.9)	0.40
Problems related to crime or the legal system	3 (7.7)	0 (0.0)	2 (18.2)	0.19	5 (7.7)	0 (0.0)	0.15
Other: personal history of self-harm	13 (33.3)	3 (21.4)	3 (27.3)	0.31	19 (29.2)	5 (13.2)	0.06

* Statistically significant difference.

The group of psychosocial stressors reported with the highest frequency for the study sample was relational problems (64.1%), a category that reflects problems related to family upbringing or the primary support group, and which may include stressors such as high expressed emotion, bereavement, and separation or divorce. Slightly more than half of the patients (55.3%) faced educational and occupational stressors, such as problems adapting to or performing in an educational environment, unemployment and job dissatisfaction. A history of abuse and neglect was reported by 28.2% of the patients, which includes a history of being abused, neglected or mistreated as a child or during an intimate adult relationship. Almost a quarter of the patients (24.3%) reported other problems related to social environment, such as perceived experiences of social exclusion, discrimination, problems with acculturation and coping with different phases of life. Only five patients reported problems related to crime or interaction with the legal system. No stressors related to housing or economic problems were recorded in the clinical files. In the category of ‘other’ conditions that warrant clinical attention, the only frequently recorded stressor was a personal history of self-harm, and this was reported by almost a quarter (23.3%) of the patients. The only significant difference between the three main single anxiety diagnosis patients was regarding other problems related to the social environment (*p* = 0.02), which occurred in nearly half of the SAD group, a quarter of the GAD group and none of the panic disorder group. Personal history of self-harm occurred more frequently in those with single anxiety diagnosis than those with multiple anxiety diagnoses (*p* = 0.06). No other significant differences were found between the single anxiety and multiple anxiety diagnoses groups.

## Discussion

To meaningfully compare the results of this study with previous local research, foremost of which is the SASH study, it should be kept in mind that the SASH study was a community prevalence study, whilst this study focused specifically on tertiary-level psychiatric outpatients. Within this tertiary psychiatric setting, anxiety disorders were diagnosed in 15.8% of all active mental healthcare patients at the FSPC outpatient unit. The large majority of persons with anxiety disorder diagnosis in this study were females, a fact that correlates with the gender association found by the SASH study.^1^ However, the SASH study demonstrated an association between anxiety disorder and primary level of education,^1^ which was not found in this study as most of the participants (59.2%) had attained at least 12 years of formal education. A striking difference between the findings of this study and data provided by the SASH study^1^ pertains to the diagnostic rates of the different disorders, especially agoraphobia. The SASH study^1^ reported that agoraphobia without panic was the most prevalent anxiety disorder and one of the three most prevalent individual lifetime mental disorders, whereas in this study agoraphobia occurred, with the exception of one case, only within the context of panic disorder. This finding may in part be explained by the possibility that prior to the recent formal separation from the diagnosis from panic disorder with the implementation of the DSM-5,^8^ clinicians at the FSPC outpatient unit coded agoraphobia as part of panic disorder, even in the absence of clinically significant panic data. Alternatively, the SASH study’s findings on agoraphobia without panic may reflect an overestimation of agoraphobia in the general population. Other possibilities for the less than expected rates of agoraphobia observed in this study include possible difficulties in accessing mental healthcare services because of functional impairment or poor availability of services, or poor help-seeking behaviour among those suffering from agoraphobic fears in the absence of panic symptoms, as they may not recognise the need for treatment.^1^

The high prevalence of comorbidity found in international literature^11^ between anxiety, depressive and substance use disorders was also observed in the current study. This high level of comorbidity may be because of overlapping symptomatology between depressive and anxiety disorders. Our findings suggest that in this tertiary psychiatric setting, anxiety disorders are rarely diagnosed in the absence of another psychiatric disorder. This may suggest that persons who suffer from anxiety disorders in the absence of other psychopathologies might receive treatment at primary or secondary level of care. Another finding pertaining to comorbidity of the anxiety disorders that also warrants further investigation is that patients with multiple anxiety disorders also had higher rates of a comorbid substance-related disorder when compared to those with only one anxiety disorder. It should be noted that patients whose only anxiety disorder diagnosis was recorded as substance- or medication induced were not considered in this study. Therefore, the full impact of substance use on anxiety symptoms is not reflected by these results. A surprising finding related to the low rates of comorbidity with personality disorders. Given the expected high comorbidity rates described in international literature,^12^ this finding may reflect the possible under-diagnosis of either personality disorders among patients who present with anxiety disorders or anxiety disorders among persons diagnosed with personality disorders at the study site. The possibility of undiagnosed personality pathology could further be reflected by the finding that nearly a quarter of patients, most of whom had a single anxiety disorder diagnosis, also had a personal history of self-harm that warranted clinical attention. Self-harm has been associated with poorer treatment outcomes.^7^ Therefore, further studies should be conducted to explore the incidence and diagnostic implications of self-harm among patients with anxiety disorders.

In this study, patients with anxiety disorders were found to experience different types of psychosocial stressors, with significant relational problems occurring among the majority of patients. This finding is especially significant given the suggested association between high expressed emotions in the family environment with poor treatment response in anxiety disorders.^7^ Educational or occupational problems were recorded for more than half of the patients. Just over a quarter of patients experienced abuse and neglect, and other problems of the social environment were experienced by just under a quarter of the patients included in this study.

### Study limitations

A limitation of the study is the lack of generalisability of the findings to other psychiatric settings because of the demographic profile of the patients and the fact that patients were not re-assessed through clinical interview to confirm diagnosis during the course of this study. Information may not have been captured correctly or adequately in the clinic files from which the data for this study were obtained. Furthermore, methodological problems, such as an unfamiliarity on the part of the researchers with the abbreviations encountered in the clinical files as well as unclear handwriting and missing information in some clinical files, place further limitations on the study despite the use of mitigation strategies, such as continuous consultation with a clinician at the FSPC outpatient unit.

## Conclusion and recommendations

This study provides additional information about the diagnostic and psychosocial profiles of adult patients receiving treatment for anxiety disorders at a South African tertiary psychiatric facility. Certain aspects in particular were highlighted by this study. The high rates of comorbidity found, especially with depressive and substance use disorders, may hold two important implications for clinical practice and future research. Firstly, given the high provincial lifetime prevalence for anxiety disorders,^1^ further study is warranted to investigate if individuals with a single diagnosis of an anxiety disorder do, in fact, receive effective treatment at primary or secondary level of care. This is particularly important given the scarcity of mental health services in primary healthcare facilities in the Free State and previous research findings that the majority of South Africans with mental disorders go untreated.^13^ Secondly, the finding that a little over one-third of patients receiving treatment for anxiety disorders also had a substance-related disorder diagnosis necessitates an evaluation of how the treatment and rehabilitation of substance use disorders are integrated into psychiatric services for these patients, as it is not known at present how frequently these mental healthcare users utilise substance rehabilitation programmes as part of inclusive tertiary-level intervention procedures. Substance withdrawal and rehabilitation services are under-resourced in the Free State and, at the study site, only one staff member is assigned specifically to the rehabilitation programme. The results of this study highlight the importance of integrated substance-related interventions within specialised mental healthcare services.

Further recommendations include investigating the practices regarding assessment of comorbidity of anxiety and personality disorders at the study site, as this may hold important implications for prognosis and treatment.^14,15^ This is further underscored by the finding relating to a personal history of self-harm among nearly a quarter of patients with anxiety disorders in this study. In addition, the low rates of occurrence of agoraphobia in the absence of panic disorder relative to what has been found for the local general population warrant consideration of diagnostic practices, in particular the implementation of the DSM-5 system, at the study site to ensure that existing tertiary mental healthcare users are sufficiently screened for agoraphobia. The lower than expected rates of agoraphobia, furthermore, warrant investigation into its prevalence in existing community mental health services in order to determine if agoraphobic individuals primarily receive treatment at lower levels of care and what their treatment outcomes are. Finally, the results further suggest that specific psychosocial interventions may need to be included in the management of patients with anxiety disorders. The psychosocial stressors most frequently experienced by patients – namely, relational problems, educational and occupational stressors, a history of abuse and neglect, and other problems related to social environment – should be the focus of further research in order to gain a better understanding of the impact they might have on the clinical management of these patients.
